# Matrix Metalloproteinase 2: an important genetic marker for cholesteatomas

**DOI:** 10.1016/S1808-8694(15)31122-8

**Published:** 2015-10-20

**Authors:** Douglas Salmazo Rocha Morales, Norma de Oliveira Penido, Ismael Dale Coltrin Guerreiro da Silva, João Norberto Stávale, Arnaldo Guilherme, Yotaka Fukuda

**Affiliations:** 1 PhD and M.S. Assistant Physician – University Hospital - USP; 2 PhD and M.S. Assistant, Head of the Interdisciplinary Otology Department of ENT/HNS - EPM Unifesp; 3 PhD. Associate Professor – Department of Molecular Gynecology - EPM Unifesp; 4 PhD and M.S. Assistant Professor – Department of Pathology - EPM Unifesp; 5 PhD and M.S. Professor – Department of Otorhinolaryngology - EPM Unifesp; 6 PhD and M.S. Associate Professor – Department of ENT/HNS EPM Unifesp e do Hospital do Servidor Público Estadual de São Paulo

**Keywords:** cholesteatoma, metalloproteinase

## Abstract

**Summary:**

Aim: This study is to determine the MMP2's presence in cholesteatomas and whether complicating cholesteatomas show a higher immunohistochemical expression of matrix metalloproteinase 2. Cholesteatoma produces enzymesthat cause bone erosion like Matrixmetalloproteinase 2 (MMP2).

**Material and Methods:**

We analyzed the expression of MMP2 in invasive (causing complications) compared to latent cholesteatomas (not causing complications). A crosssectional study with nineteen slides and paraffin blocks of cholesteatomas derived from mastoidectomies were located and processed, including 8 invasive and 11 latent cholesteatomas. Immunohistochemical thecnique was empregated to MMP2.

**Results:**

The results are expressed as 0, + (to low), ++ and +++(high) according to the quantity and color of the immunohistochemical staining of MMP2. Higher expression of MMP2 was observed in 7 (87.5%) of the 8 invasive cholesteatomas. With respect to latent cholesteatomas, higher expression of MMP2 was observed in 27.3% (3 cases), with Fisher's exact test indicating a significant difference (p=0.015).

**Conclusions:**

Cholesteatoamas express MMP2 and Invasive cholesteatomas had high MMP2 compared to latent cholesteatomas.

## INTRODUCTION

A daily cholesteatoma approach is part of most otorhinolaryngologists' routine. Notwithstanding, we still lack a genetic marker that predicts its complication potential. The marker hereby chosen is called Matrix Metalloproteinase 2 (MMP2). Metalloproteinases are enzymes capable of causing bone erosion, especially the MMP2, which has become object of correlation with cholesteatomas. Based on samples of more and less aggressive cholesteatomas (given clinical signs and the complications brought about by the disease), except for congenital cholesteatomas and patients with acquired cholesteatomas in less than 14 years (average time length taken by the cholesteatoma in order to bring about complications), we tried to show whether or not there is a correlation between the expression of type 2 metalloproteinase (MMP2) and the cholesteatoma's aggressiveness.

Our goal with the present investigation is to observe if cholesteatomas in general produce matrix metalloproteinase 2 (MMP2), and if more aggressive cholesteatomas produce a higher quantity of MMP2.

### Cholesteatoma and complications

According to Ribeiro Pereira^1^, a cholesteatoma is an epidermal cyst, made up of keratinized epidermal tissue, capable of migrating and eroding adjacent structures, frequently found in temporal bones with history of chronic otitis media. The epidermoid cyst bears an external matrix formed by stratified keratinized squamous epithelium over a secondary matrix of fibro-connective tissue (connective tissue bearing collagen elastic fibers, a mash of fibers, fibroblasts, lymphocytes, histiocytes and plasmocytes). From the histological stand point, the epithelium is similar to the epidermis, and its four basic layers can be seen (basal, squamous, granulous and cornea). Such matrix sheds keratin lamellae to within the space limited by the epidermal sac, filling and distending it. According to Soldati and Mudry^2^ the cholesteatoma was first described in 1829, by Curvalhier, as a tumor-like process, and was thus called because of the cholesterol found inside, as it was also described by Muller in 1830. There is a report that in 1683, Duverney described a temporal bone tumor that may very well have been a cholesteatoma. In 1855, Virchow thought the cholesteatoma was a tumor that increased in size because of mesenchymal cell metaplasia towards epithelial ones. Gruber, Wendt and von Troelsch, in 1868, described the cholesteatoma as the result of tympanic membrane cell metaplasia into malpighia epithelium. In 1869, Politzer stated that the cholesteatoma was but a glandular neoplasia of the middle ear mucosa. Bezold and Habermann, in 1889, published that the cholesteatoma was the result of an epidermal migration from the external auditory canal towards the middle ear cavity via a marginal perforation after an episode of chronic or acute otitis media. In 1980, Plester^3^ stated that hereditary or genetic factors influenced the degree of mastoid pneumatization, by skull base alterations that interfered in the Eustachian tube anatomical position and patency, thus altering mastoid pneumatization. In cholesteatoma patients, the angle formed by the posterior glenoid process and the posterior border of the hard palate is significantly smaller when compared to patients without cholesteatoma. The epipharynx configuration is considerably smaller in cholesteatoma patients. Albino, Kimmelman and Parasier^4^ analyzed studies about the cholesteatoma ultrastructure and concluded that: (1) cholesteatomas do not present inherent genetic instability, differently from malignant neoplasias; (2) The induction of hyperproliferative cells in the cholesteatoma epidermal base implies an idiopathic response potential, originated from internal events and external stimuli, through cytokines produced by inflammatory cells. (3) Bacteria may initiate the cholesteatomatous process. (4) So far, there are no molecular or cellular differences among the different cholesteatoma types (primary, secondary, recurrent, congenital). According to Caldas and Caldas Neto^5^, the expansive and destructive capacity inherent to cholesteatomas may compromise the middle ear ossicular chain, eventually bringing about erosions to the inner ear bony protection, facial nerve, meninges and lateral sinus, causing irreversible lesions and remote infections that may leave patients with permanent functional disabilities or even death. According to Morales, Cervantes and Testa^6^, who in 2001 described the possibility of cholesteatomas producing intra and extracranial complications (neck and face) because of erosions caused to the adjacent temporal bone and the secondary infection induced by this very erosion. Extracranial complications are: labyrinthitis, deafness, peripheral facial paralysis, mastoiditis (Bezold type, squamous-zygomatic and Mouret type) and middle ear ossicular chain destruction (malleus, incus and stapes). Intracranial complications are: meningitis, meningoencephalitis, cerebral or cerebellar empyema, cerebral or cerebellar abscess and lateral sinus thrombosis. Voegels, Garcia, Bogar, Miniti and Bento^7^ studied 14 patients with cholesteatoma-induced semicircular canal fistula, with anacusis as a major disease complication. In 2001, Penido and Fukuda^8^ studied 25 cases of brain abscess (cerebral or cerebellar) of otological origin and concluded that most of the times the cause is a cholesteatomatous chronic otitis media, and besides draining the encephalic abscess, the mastoid should be opened in order to clear the infectious process. In 2003, Cruz, Kasse and Leonhart^9^ presented a retrospective study that revised 84 (eighty-four) ears with chronic otitis media, 41 (forty-one) non-cholesteatomatous and 43 (forty-three) cholesteatomatous, and observed that canal wall down mastoidectomy precludes disease recurrence (4%); while the canal wall up procedure yielded a 10% recurrence rate. In 2003, Testa, Vicente, Abreu, Benbassat, Antunes and Barros^10^, carried out a retrospective study involving 206 facial nerve decompressions in order to treat peripheral facial paralysis and observed 10 cases (4.85%) of cholesteatomas as the cause for such complication. In 2005, Penido, Borin, Iha, Suguri, Onishi, Fukuda and Cruz^11^, in a retrospective study that analyzed therapeutic options in 33 (thirty-three) patients with otitis media causing intracranial complications, found the cholesteatoma to be the most common etiology of such complications (26 cases, or 79%).

### Cholesteatomas and the Matrix Metalloproteinase 2

In 1996, Schonermark, Mester, Kempf, Blaser, Tscheche and Lenarz^12^ found that tissue invasion by cholesteatomas is related to the MMP2, which had also been described in 1996, by Gohlke, GomisRuth, Crabbe, Murphy, Docherty and Bode^13^, then called Gelatinase A, that bears a non-catalytic C terminal domain that bears a homologous sequence and haemopexin. Deslogue et al.^14^ (1997), in a study called: Human cholesteatoma DNA analysis, noticed the presence of euploid DNA in cholesteatomas. Banerjee, James and Narula^15^ (1997) by means of the Western blotting technique, showed the presence of metalloproteinase 2 (MMP2) in cholesteatomas. Metalloproteinase 2 (MMP2) or Gelatinase A or 72 Kda collagenase is a protein induced in the cell nucleus and is related to properties such as tumor progression - including growth, invasion, metastasis and angiogenesis (blood vessel growth), agreeing with Sun and Hemler^16^ (2001), who recognized the extracellular interactions of the MMP2, acting as proteolytic enzymes capable of degrading tissue connective components. Zhu, Xie and Wang^17^ (2001), in a study called “Matrix metalloproteinase^2^,^9^ expression in middle ear cholesteatoma and cancer”, by means of immunehistochemical methods for MMP 2 and 9, used 36 cholesteatomas and 10 external ear skin fragments and 16 middle ear cancer fragments, observed a direct relation between cholesteatomas and MMP2 and MMP9, thus concluding that this disorder between metalloproteinases and their inhibitors was one of the reasons for bone resorption in middle ear cancer and cholesteatoma cases. Bernal Sprekelsen, Ebmeyer, Anonopoulos, Borkowiski and Sudhoff^18^ (2001) stated in their study called: “Middle ear cholesteatoma base membrane alterations “, that the cholesteatoma epithelium is characterized by the lack of keratinocyte regulation, followed by the destruction of ossicles and other temporal bone parts. By means of immunohistochemistry, they noticed that metalloproteinases and the basic fibroblast growth factor may explain both destructive and proliferative middle ear cholesteatoma activities. In an experimental study with gerbils (Mongolian squirrel) (published in 2002), they tested the MMP2 topical inhibitor, Ilomostat, observing whether or not there is any relation with the formation of atelectasis on the tympanic membrane of these animals, and no statistical difference was observed between the group that received the drug for eight weeks (6 animals) and the control group (7 animals); it could not be stated for sure that the same thing could correlate to human cholesteatomas. In 2003, Wilmoth, Schultz and Antonelli^19^ studied 48 gerbils and observed that metalloproteinases are Zinc (Zn)-linked enzymes, and represent elements capable of destroying the extracellular matrix. According to the authors, MMPs are known for participating in normal collagen metabolism physiology, but are also associated to pathological absorptions, participating in chronic inflammatory skin processes, tumors and metastasis in the extracellular matrix. MMP's activities are regulated by metalloproteinase inhibitors and the breakage of such harmony may trigger a pathological process. In this study, the tympanic membranes of the animals were placed within cultures with bacterial toxins (lipopolysaccharides and alpha tumoral necrosis factor), noticing that their chains started to Express a greater amount of metalloproteinases (MMP2), which could contribute to the bone resorption process.

In a study published in 2004, Gaiotto et al.^20^ described that metalloproteinase 2 (MMP2) expression in normal uterine cervix, intraepithelial neoplasia and uterine cervix squamous cell carcinoma was gradually higher according to the degree of malignity and infiltration. The study shows that MMP2 is an enzime produced in the cell nucleus and its expression is related to its capacity to infiltrate and invade adjacent tissue. While that paper was being published, the concept of MMP2 tissue invasion was used in relation to cholesteatomas in this study.

## METHODS

This research project has been submitted to the Ethics in Research Committee of the Federal University of São Paulo - São Paulo Hospital, and approved under protocol # 0322/03. Mastoidectomies were carried out in the São Paulo University Hospital-Federal University of São Paulo in the last 10 years, due to middle ear cholesteatomas. We investigated patients' charts, looking for cholesteatoma-related complications (mastoiditis, labyrinthitis, peripheral facial paralysis, encephalitis, meningitis, lateral sinus thrombophlebitis, cerebral abscess, cerebellar abscess), as an indication of worse prognosis (more aggressive). Patients below 14 years of age and those with congenital cholesteatomas were taken off the sample, thus reducing sample size, together with technical difficulties related to the storage of paraffin blocks, slide labeling and immunohistochemical reactions. Treatment is primarily surgical, in other words, mastoidectomy, according to a study published by Cruz, Kasse and Leonhart, when fragments are removed for pathology and immunohistochemical analysis. We used 19 cholesteatoma paraffin blocks (8 of the aggressive type). We then proceeded with the immunohistochemical analysis in order to detect MMP2 expression. The paraffin blocks were cut in slides measuring from 3 to 4 micrometers, and assembled in previously labeled slides at 4%. The paraffin was then removed from the slides using an oven at 57°C during the night, and they were immersed in a xylol bath in the following duration sequence: 30, 5 and 5 minutes more. They were then hydrated in ethylic alcohol in decreasing concentrations of 100%, 80% and 70%, respectively; each one lasting 5 minutes, and were then washed in tap water.

On the following step, we carried out antigenic recovery in a microwave oven (maximum power output of 700 Watts), and the slides were placed in a sodium citrate buffer solution, pH=6.0, during 45 minutes, and were then washed in tap water. Endogenous peroxidase block was carried out with four 5 minute baths in 20 volumes hydrogen peroxide (H2O2), followed by distilled water wash and PBS (saline solution buffered with phosphates), pH= 7.4 7.6. We used antibody MMP2/72kDa Collagenase IV (Human, Mouse, Rat and Cow) Paraffin diluted in BSA 1% (bovine albumin serum) at 1: 150 titration. All the slides were then placed in a single chamber at 4°C for the night. The material was washed with PBS buffer and incubated with the biotinilated secondary antibody. Washed with PBS solution.

In order to develop the reaction we used a chromogenic substrate, the DAB solution (33-Diaminobenzidine) at the rate of 0.06g for 100 ml of PBS and 1 ml of H2O2 at 20 volumes, for 5 minutes at 37°C. As a final product of such reaction, we observed in the microscope the shape of a dark brownish precipitate. The material was then washed in tap water and counter-dyed with “Hematoxylin Mayer” for 3 minutes. Following, it was submitted to 3 baths of xylol for diafinizing. The slides were then, finally, assembled with micro slides and Entellan®.

Slide reading was carried out under light microscopy with a 200 and 400 times magnification (Olympus BX 40 microscope), by two independent observers (one of them is a professor of pathology and the other is the researcher himself). We observed whether or not MMP2 was present in the cholesteatoma samples and the intensity of MMP2 coloring. The result was expressed in 0, +, ++ and +++, according to the amount of MMP2 and color intensity found (attenuated or absent, beige, brown, dark brown, respectively).

## RESULTS

**Figure 1:**
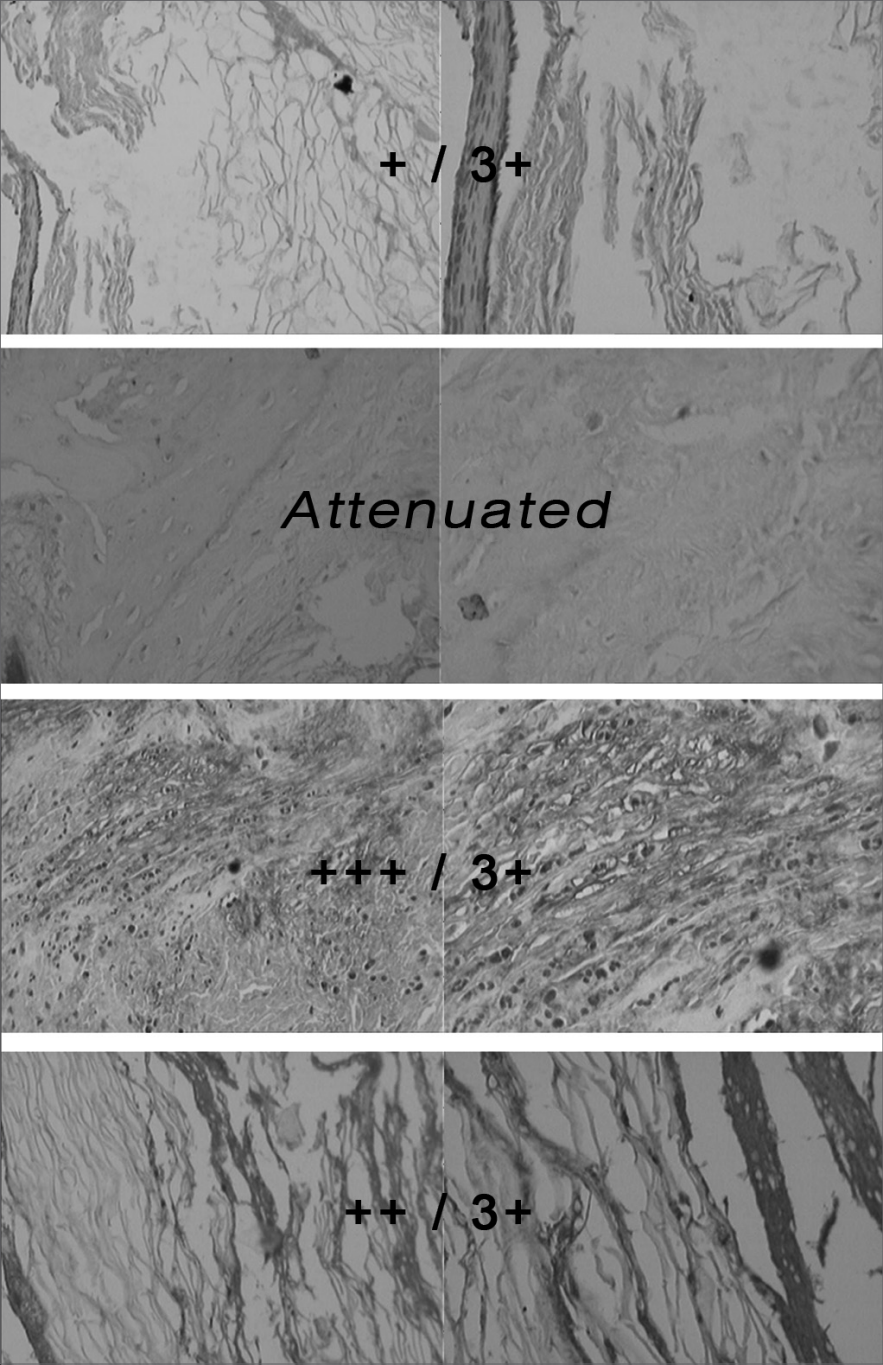
Picture of cholesteatoma slides submitted to MMP2 immunohistochemistry reaction, presenting different intensities, read under light microscopy, with 200 and 400x magnifications. - attenuated and +/3+: Less MMP2 expression. ++/3+ and +++/3+: Greater MMP2 expression.

The 19 cholesteatoma slides (8 of them were invasive) underwent an immunohistochemical technique for MMP2 detection, causing the reading of a qualitative MMP2 presence in the outer portion of the cholesteatoma. The reading of the MMP2 immunohistochemical expression in the cholesteatoma slides was expressed in values of: 0 (Attenuated), + (Mild), ++ (Moderate) and +++ (Intense). (Figure 1).

We can see that in the nineteen cases analyzed, fifteen presented mild, intense or moderate MMP2 expression, making up a total of 78.9% of the initial sample and the remaining were attenuated, though present. Of the cholesteatomas that did not present clinical complications, eleven of the nineteen studied, two of them expressed attenuated MMP2 (18.1%), six expressed MMP2 with mild intensity (54.6%), three expressed MMP2 with moderate intensity (27.3%). Of the cholesteatomas that presented complications, eight of the nineteen: one had attenuated MMP2 expression (12.5%), three expressed MMP2 moderately (37.5%), and four strongly expressed MMP2 (50%). (Chart 1, Table 1 and Graph 1).

**Chart 1:** Cholesteatoma slides and their association with patient's gender, age, slide number, MMP2 expression intensity and complication (when it happened). - MMP2: matrix metalloproteinase 2.

Initials	Gender	Age	Slide	MMP2 reading	Complication
D.G.P.V.	Male	17 years	B02 14933	++	Lateral sinus thrombosis
R.M.D.S.S.	Male	18 years	B00 07723	++	Left temporal lobe abscess
J.C.D.S.	Male	24 years	B02 28657	++	Meningitis
O.C.D.S.	Male	37 years	B98 09318	+++	Left temporal bone abscess and meningitis
J.P.D.S.	Female	22 years	B01 18345	+++	Sigmoid sinus thrombosis and meningitis
A.P.D.S.	Female	30 years	B02 35536	+++	Sigmoid sinus thrombosis
A. A. S.	Female	35 years	B03 32668	+++	Labyrinthitis and erosion of the facial nerve canal
V.L.B.D.S	Female	32 years	B04 04518	0	Meningitis and peripheral facial paralysis
W.F.P.	Male	16 years	B03 5856	0	No complications
J.D.R.	Male	17 years	B92 408	++	No complications
R.P.	Male	19 years	B03 2724	0	No complications
S.S.L.	Male	66 years	B03 24412	+	No complications
R.D.J.S.	Male	21 years	B03 4079	+	No complications
L.C.S.	Female	15 years	B03-521	++	No complications
E.N.D.	Female	26 years	B03 01011	+	No complications
S.H.V.	Female	32 years	B04 4205	++	No complications
L.G.G.	Female	50 years	B94 7353	+	No complications
S.M.S.B.	Female	38 years	B03 670	+	No complications
M.M.C.D.S.	Female	62 years	B03-1132	+	No complications

**Table 1:** Shows the number of cholesteatoma slides, associating the complicated and non-complicated ones to gender, age, MMP2 immunohistochemical expression, with the corresponding rate of significance. - p: significance rate less than 0.05.

	Cholesteatoma		
	Latent (n=11)	Invasive (n=8)	P
Males	5 (45,5%)	4 (50%)	
Age (years)	32,9 +-18,7	26,9 +-7,7	
	26,0 (17-50)	27,0 (19-34,3)	
MMP2 expression			
0 ou +	8 (72,7%)	1 (12,5%)	# 0,015
++ ou +++	3 (27,3%)	7 (87,5%)	

# statistical test of significant Fisher.

**Graph 1:**
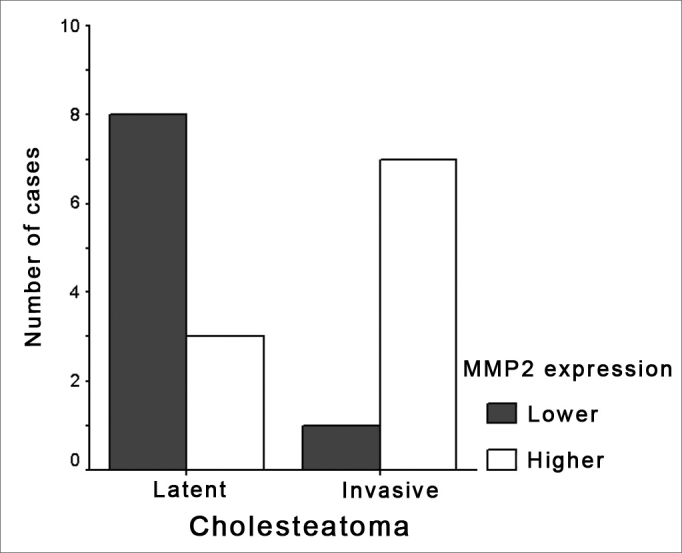
Association between cholesteatomas (invasive and latent) and MMP2 immunohistochemical expression (greater and lesser). - MMP2: matrix metalloproteinase 2. Greater: greater (+++ and ++) MMP2 expression in the cholesteatoma. Lesser: lesser (attenuated and +) MMP2 expression in the cholesteatoma.

## DISCUSSION

It is conceptually accepted that each and every cholesteatoma bears the potential to cause intra and extracranial complications, as stated by Penido et al. (in 2004) in a retrospective study that analyzed therapeutic options for cholesteatoma treatment in 33 patients with intracranial complications caused by otitis media, observing that the cholesteatoma is the most common basic etiology of these complications (26 cases or 79%); Caldas and Caldas Neto in 1988; and Morales, Cervantes and Testa in 2001, described the intracranial (meningitis, encephalitis, cerebral abscess, cerebellar abscess and empyema) and extracranial (neck and face: peripheral facial paralysis, labyrinthitis, mastoiditis: Bezold, Mouret, squamous-zygomatic and cervical abscesses) complications. According to Plester (1980), hereditary or genetic factors influence the degree of mastoid pneumatization, causing narrower angles at the attic, a determining factor for the generation of more aggressive cholesteatomas; this by itself seems to be enough to explain precisely the erosion caused by cholesteatomas to the adjacent bone tissue in some cases and not in others, suggesting that there must be some intrinsic cholesteatoma expression factor that would explain why the erosion caused by some cholesteatomas is greater (and thus brings complications) than by others.

Thus, from an universe of studies about collagenases, growth inhibitors and cholesteatoma prolipheration, osteolytic enzymes, bacterial toxins such as lipopolysaccharides and tumor necrosis alpha factor, in 1996, Schonermark, Mester, Kempf, Blaser, Tscheche and Lenarz studied cholesteatomas and metalloproteinases, which are proteolytic enzymes responsible for bone homeostasis, present in greater quantities in osteolytic inflammatory diseases and observed the presence of MMP2, MMP9 and MMP3 in the basal and suprabasal cells of cholesteatomas. The results of this study indicate the possibility that members of the metalloproteinase family may activate bone invasion mechanisms in cholesteatomas.

Back in 1998, Banerjee, James and Narula demonstrated that MMP2 and MMP9 were present in cholesteatomas and in the external auditory canal skin. Apparently, such fact would hamper research, since the normal external auditory canal tissue also had the enzime deemed important in the explanation of the bone erosion mechanism.

In 2001, Bernal Sprekelsen, Ebmeyer, Anonopoulos, Bordowiski and Sudhoff, insisted and noticed that metalloproteinases and the fibroblast basic growth factor could explain cholesteatomas' destructive and proliferative activities.

Also in 2001, Zhu, Xie and Wang, published a study involving cholesteatomas and middle ear cancer, stating that the disorder between metalloproteinases and their inhibitors was one of the reasons behind middle ear cholesteatoma and cancer causing bone resorption. Therefore, the assumption that MMP2 was expressed in cholesteatomas and the external auditory canal skin still had its worth; that fact that they were raising is that cholesteatomas stimulated by infectious factors produce more MMP2 than the external auditory canal skin, which was then proved by Wilmoth, Schultz and Antonelli, in 2003, who observed in an experimental study that tympanic membranes of lab animals (gerbils) placed in a culture with bacteria toxins (lipopolysaccharides and tumor necrosis alpha factor), expressed greater quantities of metalloproteinases. From this moment on, a question arised: what comes first, the infection or the metalloproteinase? Answer: the metalloproteinase, since it is embedded in the cell genetic code. But then, why not all cholesteatomas cause bone erosion? Why only some complicate? Because the expression is related to a mismatch between MMP activators and inhibitors; thus, some cells have a genotype with a greater capacity to express MMP2 when compared to others, bearing in mind that infection is a potent stimulus for metalloproteinase expression by the cholesteatomatous cell. However, does a cholesteatoma complicate when it expresses more metalloproteinase or is it due to the fact that it harbors a more virulent infection (intense)? It is, in fact, a hard question to answer. Nonetheless, if cholesteatomas produce more metalloproteinase, it will ultimately destroy more bone tissue, thus bringing forth complications.

In Chart 1, of the 11 patients with latent cholesteatomas, 2 had no MMP2 expression (0), six patients (6) had MMP2 expression of +, and 3 patients expressed MMP2 as ++. In Chart 1, we may also notice that of eight (8) patients with invasive cholesteatomas, only one (1) had a lesser MMP2 expression (0); the remaining patients with invasive cholesteatomas manifested a higher MMP2 expression (++ and +++). (Figure 1).

These figures were statistically analyzed by the Fisher Exact Test, presenting significant results with p= 0.015, in other words, cholesteatomas that do complicate present a “++ or +++” immunohistochemical MMP2 expression, when compared to latent cholesteatomas that bear MMP2 immunohistochemical expression of “0 or +”.

With the use of metalloproteinase inhibitors (ilomostat), as mentioned in the experimental study carried out by Lehman, Wilmoth, Prevatt, Schultz and Antonelli (2002), and that of Wilmoth, Schultz and Antonelli (2003), it is suggested that cholesteatomas would cause less erosion to the adjacent bone tissue, and this may help prevent intra or extracranial complications (neck and face).

Another assumption we may suggest is that when we operate on a patient with cholesteatoma, if we carry out an MMP2 immunohistochemical reaction, we may increase the prognostic accuracy on the operated lesion. A greater challenge would be to study other enzymes from the family of metalloproteinases, in order to better understand the genetic content of a cholesteatoma, and thus be able to individualize its genetic characteristics for a given patient, and thus have a more accurate prognosis on its proliferation and erosive capacity.

## CONCLUSION

This study allows us to state that:

1. All cholesteatomas express MMP2, but 84% of the cholesteatomas investigated express MMP2 lightly, moderately and intensely; and 16% express attenuated MMP2 (this is similar to what happens to other tissues).
2. Cholesteatomas that cause complications have a MMP2 immunohistochemical expression that is more intense, proven by the Fisher Exact Test, with p=0.015 (significant), when compared to cholesteatomas that do not cause complications

## References

[1] Ribeiro FAQ, Pereira CSB, Campos CAH, Costa HOO (2002). Otite Média Crônica Colesteatomatosa. 1..

[2] Soldati D, Mudry A (2001). Knowledge about cholesteatoma from the first description to the modern histopathology.. Otol Neurotol.

[3] Plester D (1980). Hereditary factors in chronic otitis with cholesteatoma.. Acta Otorhinolaryngol Belg.

[4] Albino AP, Kimmelman CP, Parisier SC (1998). Cholesteatoma: a molecular and cellular puzzle.. Am J Otol.

[5] Caldas N, Caldas Neto N (1988). Considerações sobre colesteatomas residuais e iatrogênicos.. Rev Bras Otorrinolaringol.

[6] Morales DSR, Cervantes O, Testa JRG (2001). Mastoidites e suas complicações: relato de quatro casos.. Compacta.

[7] Voegels RL, Garcia M, Bogar P, Miniti A, Bento RF (1994). Fístula perilinfática devido à colesteatoma: estudo de 14 casos.. Rev Bras Otorrinolaringol.

[8] Penido NDO, Fukuda Y (2001). Abscesso encefálico otogênico.. Rev Bras Otorrinolaringol.

[9] Cruz OLM, Kasse CA, Leonhart FD (2003). Efficacy of surgical treatment of chronic otitis media.. Otolaryngol Head Neck Surg.

[10] Testa JRG, Vicente ADO, Abreu CEC, Benbassat SF, Antunes ML, Barros FA (2003). Colesteatoma causando paralisia facial.. Rev Bras Otorrinolaringol.

[11] Penido NDO, Borin A, Iha LCN, Suguri VM, Onishi ET, Fukuda Y, Cruz OLM (2004). Intracranial complications of otitis media: 15 years of experience in 33 patients.. Otolaryngol Head Neck Surg.

[12] Schonermark M, Mester B, Kempf HG, Blaser J, Tschesche H, Lenarz T (1996). Expession of matrix metalloproteinases and their inhibitors in human cholesteatomas.. Acta Otolaryngol.

[13] Gohlke U, GomisRuth FX, Crabbe T, Murphy G, Docerty A J, Bode W (1996:8). The Cterminal (haemopexinlike) domain structure of human gelatinase A (MMP2), structural implications for its function.. FEBS Lett.

[14] Desloge RB, Carew JF, Finstad CL, Steiner MG, Sasson J, Levenson MJ, StaianoCoico L, Parisier SC, Albino AP (1997). DNA analysis of human cholesteatomas.. Am J Otol.

[15] Banerjee AR, James R, Narula AA (1998). Matrix metalloproteinase2 and matrix metalloproteinase 9 in cholesteatoma and deep meatal skin.. Clin Otolaryngol.

[16] Sun J, Hemler ME (2001). Regulation of MMP1 and MMP2 Production through CD147/Extracellular Matrix Metalloproteinase Inducer Interactions.. Research.

[17] Zhu W, Xie Y, Wang P (2001). Expression of matrix metalloproteinasa 2 9 in cholesteatoma and middle ear cancer.. Zhonghua Er Bi Yan Hou Ke Za Zhi xue hui Beijing.

[18] Gaiotto MA, Focchi J, Ribalta JL, Stávale JN, Baracat EC, Lima GR, Da Silva IDCG (2004). Comparative study of MMP2 (matrix metalloproteinase 2) immune expression in normal uterine cervix intraepithelial neoplasias and squamous cells cervical carcinoma.. Am J Obstet Gynecol.

[19] Wilmoth JG, Schultz GS, Antonelli PJ (2003). Tympanic membrane metalloproteinase inflammatory response.. Otolaryngol Head Neck Surg.

[20] Bernal Sprekelsen M, Ebmeyer J, Anonopoulos A, Borkowiski G, Sudhoff H (2001). Alteraciones de la membrana basal en el colesteatoma de ouido humano.. Acta Otorrinolaringol Esp.

